# Charcot–Marie–Tooth Cavovarus Foot: Current Concepts and Emerging Strategies for Deformity Correction

**DOI:** 10.3390/jcm15166474

**Published:** 2026-08-21

**Authors:** Sung Jun Moon, Young Ki Min, Glenn B. Pfeffer, Byung Ki Cho, Chan Kang, Yea Eun Kang, Woo Jin Shin, Jae Hwang Song

**Affiliations:** 1 Department of Orthopaedic Surgery, College of Medicine, Konyang University, Daejeon 35365, Republic of Korea; konyang15@naver.com (S.J.M.); 200821@kyuh.ac.kr (Y.K.M.); tlsdnwls94@naver.com (W.J.S.); 2 Department of Orthopaedic Surgery, Cedars-Sinai Medical Center, Los Angeles, CA 90048, USA; glenn.pfeffer@cshs.org; 3 Department of Orthopaedic Surgery, Chungbuk National University Hospital, Chungbuk National University College of Medicine, Cheongju 28644, Republic of Korea; titanick25@naver.com; 4 Department of Orthopaedic Surgery, College of Medicine, Chungnam National University, Daejeon 35015, Republic of Korea; faschan@hanmail.net; 5 Department of Internal Medicine, Chungnam National University, Daejeon 35015, Republic of Korea; yeeuni2200@gmail.com

**Keywords:** Charcot–Marie–Tooth disease, cavovarus foot deformity, joint-sparing surgery, soft tissue release, osteotomy, fusion

## Abstract

Charcot–Marie–Tooth (CMT) disease is the most prevalent inherited peripheral neuropathy, characterized by progressive motor and sensory dysfunction resulting from a wide spectrum of genetic abnormalities. More than 70% of affected individuals develop cavovarus foot deformities, which impair gait mechanics, reduce functional mobility, and frequently necessitate orthotic support. The diagnosis of CMT-related foot deformity requires a comprehensive clinical evaluation, with particular emphasis on identifying muscle imbalance and assessing the function of individual musculotendinous units contributing to the deformity. Conservative management, including customized footwear and orthotic bracing, remains the first-line treatment for flexible and less severe deformities. However, surgical intervention should be considered in patients with progressive deformity, persistent symptoms, or failure of nonoperative treatment. Patients requiring surgical correction commonly present with a combination of cavovarus malalignment, muscle imbalance, soft-tissue contracture, and secondary osseous deformities. Current surgical management favors joint-sparing surgery through a combination of soft-tissue procedures, corrective osteotomies, and tendon transfer whenever feasible. Arthrodesis is generally reserved for rigid deformities that cannot be adequately corrected with joint-sparing surgery for patients with painful degenerative arthritis. When appropriately indicated, surgical reconstruction can substantially improve functional outcomes, reduce dependence on bracing, and enhance overall quality of life. This narrative review summarizes current concepts in the diagnosis and management of CMT-related cavovarus foot deformity, with particular emphasis on individualized deformity assessment and contemporary joint-sparing reconstruction.

## 1. Introduction

Charcot–Marie–Tooth (CMT) disease is the most common inherited peripheral neuropathy, characterized by progressive involvement of motor and sensory nerves. The disease leads to a broad range of clinical manifestations, including distal muscle weakness and atrophy, sensory impairment, and various musculoskeletal deformities [1]. CMT affects approximately 2.8 million individuals worldwide and is one of the most prevalent inherited neurological disorders [1,2]. More than 100 genetic subtypes of CMT have been identified. The major forms include CMT1, an autosomal dominant demyelinating neuropathy; CMT2, an axonal neuropathy; CMT4, an autosomal recessive form; and CMTX, which is inherited in an X-linked manner. Among these subtypes, CMT1A is the most common, accounting for approximately 50% of all CMT cases [1,2,3,4]. Symptoms typically manifest during childhood in patients; however, disease onset may occur later in life in a subset of individuals [5].

More than 70% of patients with CMT develop a cavovarus foot deformity [3]. CMT is the condition most commonly associated with cavus foot and should always be considered in the differential diagnosis of patients presenting with cavus deformities [6,7]. A distinguishing feature of CMT-related foot deformity, compared with other conditions associated with cavus foot such as poliomyelitis, cerebral palsy, muscular dystrophy, and stroke, is that the deformity is primarily forefoot-driven and results from plantarflexion of the first metatarsal. Consequently, the calcaneal pitch angle often remains within the normal range despite the presence of a cavovarus foot deformity [8,9]. In contrast, cavus deformities associated with other neuromuscular disorders or idiopathic etiologies are more commonly hindfoot-driven deformities. These patients typically demonstrate an increased calcaneal pitch angle, which is thought to result from weakness of the gastrocnemius–soleus complex. Although the Coleman block test is widely used to differentiate forefoot-driven from hindfoot-driven deformities, intraoperative assessment following soft-tissue release is often more reliable for determining the primary source of the deformity [8,10].

Patients with CMT-related foot deformities often experience difficulty with normal ambulation and frequently rely on orthotic devices for functional mobility. Recent studies have demonstrated that corrective foot surgery can provide significant symptomatic and functional improvement in patients who fail conservative treatment [11,12]. Such proactive surgical intervention has been reported to enable brace-free ambulation, reduce pain, and potentially delay the progression of degenerative arthritis in patients with CMT [3,13]. Although the global prevalence of CMT-related foot deformities has not been clearly established, a considerable proportion of patients presenting to orthopedic clinics with cavovarus deformity and lower-extremity weakness are likely to have underlying CMT. Nevertheless, because the clinical manifestations, diagnostic evaluation, and treatment strategies for CMT-related foot deformities have not been widely recognized and relatively few clinical studies have been conducted, management has traditionally been limited to rehabilitation therapy, shoe modifications, orthotic devices, and pain control, and some patients may experience delayed or incorrect diagnosis. Recently, the American Orthopaedic Foot & Ankle Society (AOFAS) published a consensus statement regarding the evaluation and treatment of CMT-related foot deformities, which is expected to serve as an important reference for future clinical practice [3].

In this review, we discuss the pathogenesis, clinical features, diagnostic evaluation, and current treatment strategies for CMT-related foot deformities.

## 2. Literature Search Method

A narrative review of the literature was performed to synthesize the available evidence on the diagnosis and management of CMT-related foot deformity. PubMed, Google Scholar, and Web of Science were searched from database inception through August 2026 using combinations of the terms “Charcot–Marie–Tooth,” “CMT,” “foot deformity,” “cavovarus,” “gait,” “biomechanics,” “weight-bearing computed tomography,” “orthosis,” “rehabilitation,” “tendon transfer,” “osteotomy,” “joint-sparing surgery,” “arthrodesis,” and “fusion.” Reference lists of selected publications were also manually screened to identify additional relevant studies.

Publications were considered for inclusion if they addressed CMT-related foot or ankle deformity and provided clinically relevant information regarding pathogenesis, diagnostic assessment, imaging, conservative management, rehabilitation, surgical treatment, or clinical outcomes. Original clinical studies, prospective cohorts, long-term follow-up studies, systematic reviews, and consensus statements were prioritized. Technical reports and expert reviews were included when higher-level comparative evidence was unavailable, particularly for operative techniques and treatment principles. Studies addressing non-CMT conditions were generally excluded; however, selected studies were included when they provided directly relevant methodological insights into contemporary quantitative biomechanical assessment. Titles and abstracts were initially screened according to these predefined domains, followed by full-text review of potentially relevant publications. Because this was a narrative rather than a systematic review, no formal risk-of-bias assessment or quantitative evidence synthesis was performed.

## 3. Pathogenesis

**Figure 1:**
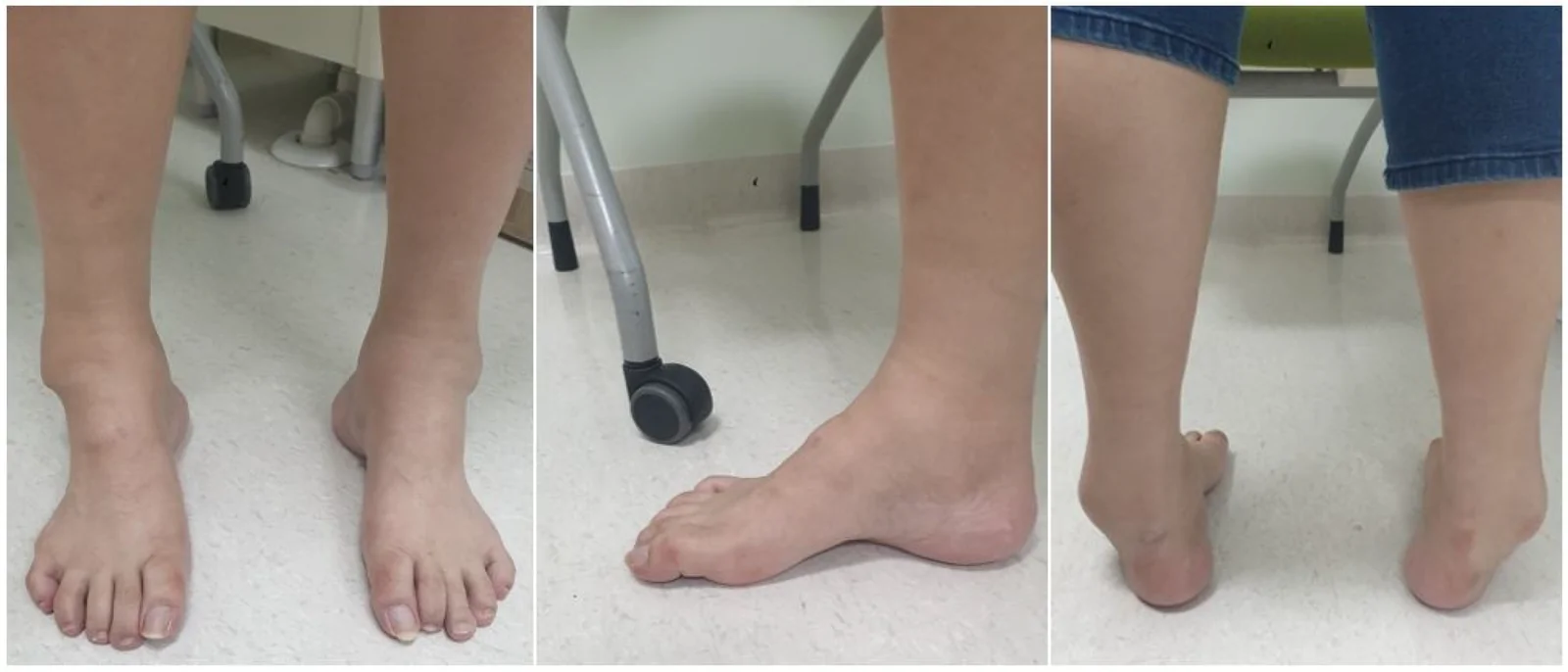
Characteristic cavovarus foot deformity showing a high arch, hindfoot varus, and claw toe deformities.

CMT foot deformity results from progressive muscle imbalance affecting the foot and ankle and, similar to other hereditary polyneuropathies, involves nearly all muscle groups. Initially flexible deformities during childhood gradually become rigid as the disease progresses. Muscle weakness and atrophy typically begin in the distal lower extremities and gradually progress proximally as the disease advances. The peroneal nerve is the most commonly affected nerve in patients with CMT. Denervation leads to weakness of the peroneus brevis and tibialis anterior, whereas their antagonists, the tibialis posterior and peroneus longus, remain relatively preserved [3]. This muscle imbalance produces hindfoot varus and plantarflexion of the first metatarsal, respectively, which together represent the fundamental pathomechanism underlying CMT cavovarus foot deformity [3]. Muscle imbalance progressively gives rise to the characteristic cavovarus deformity of the foot, which consists of hindfoot varus, plantarflexion of the first metatarsal, and inversion and supination of the midfoot (Figure 1). In the early stages of the disease, weakness of the intrinsic foot muscles leads to the development of claw toe deformities. Persistent hyperextension of the metatarsophalangeal joints places distal tension on the plantar fascia, resulting in elevation and shortening of the longitudinal arch, thereby further exacerbating the cavus deformity. As the disease progresses, weakening of the tibialis anterior compromises ankle dorsiflexion, prompting patients to compensate by increasingly recruiting the extensor hallucis longus and extensor digitorum longus. Chronic overactivity of these extrinsic extensors further accentuates the claw toe deformity [3]. Collectively, these deformities alter the normal distribution of plantar and ankle joint loading during gait, resulting in abnormal joint contact stresses that may accelerate progressive degenerative changes and ultimately lead to osteoarthritis over time.

## 4. Diagnosis and Assessment

CMT should be suspected in patients with a slowly progressive, length-dependent peripheral neuropathy characterized by distal muscle weakness and atrophy, sensory impairment, reduced deep tendon reflexes, and characteristic foot deformities, particularly when a family history of similar neurologic manifestations is present [14]. The diagnosis is primarily clinical and is supported by neurophysiological evaluation, which can help differentiate demyelinating from axonal forms, while molecular genetic testing can establish the underlying genetic subtype [14]. Once CMT has been established or strongly suspected, orthopaedic assessment focuses on the flexibility and structural characteristics of the foot deformity, the strength and balance of individual muscle-tendon units, gait impairment, and the presence of associated degenerative joint changes [3,14].

Among the various diagnostic evaluations, the most important assessment for CMT foot is a thorough physical examination of the muscle strength of each tendon contributing to the foot deformity [3]. Although nerve conduction studies, electromyography (EMG), genetic testing, and gait analysis can assist in establishing the diagnosis of CMT, the recent AOFAS consensus statement [3] indicates that these investigations are not mandatory for planning orthopedic treatment of CMT foot deformities.

### 4.1. History Taking

A detailed clinical history should be obtained to determine the age at onset of the cavovarus deformity and whether the deformity has shown a progressive course. Because CMT is an inherited disorder, the presence of similar foot deformities in first-degree relatives, including parents, siblings, or children, should also be investigated. Previous diagnostic evaluations, such as genetic testing and neurological examinations, should be reviewed, as patients with the most common genotype, CMT1A, generally exhibit a more typical and relatively milder clinical phenotype than those with other genetic subtypes [3]. In most cases, the deformity progresses over time, and patients commonly report pain during ambulation, gait imbalance, fatigue, and progressive functional limitations.

A comprehensive review of prior treatments, including both conservative and surgical interventions, is equally important. Patients with CMT foot deformities are frequently referred after having undergone surgery at other institutions; therefore, careful review of previous operative records is essential for appropriate surgical planning. Particular attention should be paid to identifying previously transferred or sacrificed tendons, as the availability of expendable tendons is a critical consideration when planning additional tendon transfer procedures.

### 4.2. Physical Examination

The physical examination should begin with careful observation of the foot during standing, sitting, and barefoot walking. In patients who routinely use an orthosis, gait should also be assessed while wearing the device to determine whether a plantigrade gait can be achieved. In addition to evaluating the severity of the cavovarus deformity, both active and passive ranges of motion of the foot and ankle should be examined, followed by a detailed assessment of the strength of each muscle-tendon unit controlling foot alignment and function.

Assessment of plantar callosities is also valuable because it reflects abnormal plantar pressure distribution during gait. In patients with CMT, hindfoot varus and midfoot supination frequently result in repetitive overloading of the lateral border of the foot. Consequently, callosities are commonly observed beneath the fifth metatarsal and other lateral plantar weight-bearing areas, providing important clues to the location and magnitude of abnormal loading.

The first step in the physical examination is to determine the flexibility of the cavovarus deformity. With the patient seated comfortably and the Achilles tendon relaxed, the examiner should assess whether the hindfoot varus and foot supination can be manually reduced to a neutral position [3]. The Coleman block test is commonly used to evaluate the flexibility of hindfoot varus and to determine whether the deformity is secondary to a plantarflexed first ray. However, in advanced CMT deformities, contracture of the tibialis posterior tendon and the medial soft tissues often prevents correction of the hindfoot even when the deformity remains potentially correctable. Consequently, a flexible deformity may be misinterpreted as rigid. Therefore, the Coleman block test should not be regarded as an absolute guide for surgical decision-making in CMT foot deformities. Instead, definitive assessment of hindfoot flexibility should be performed intraoperatively after adequate release of the tibialis posterior tendon and contracted medial soft tissues, allowing more accurate identification of the underlying deformity and appropriate planning of corrective procedures, including calcaneal osteotomy [3].

**Figure 2:**
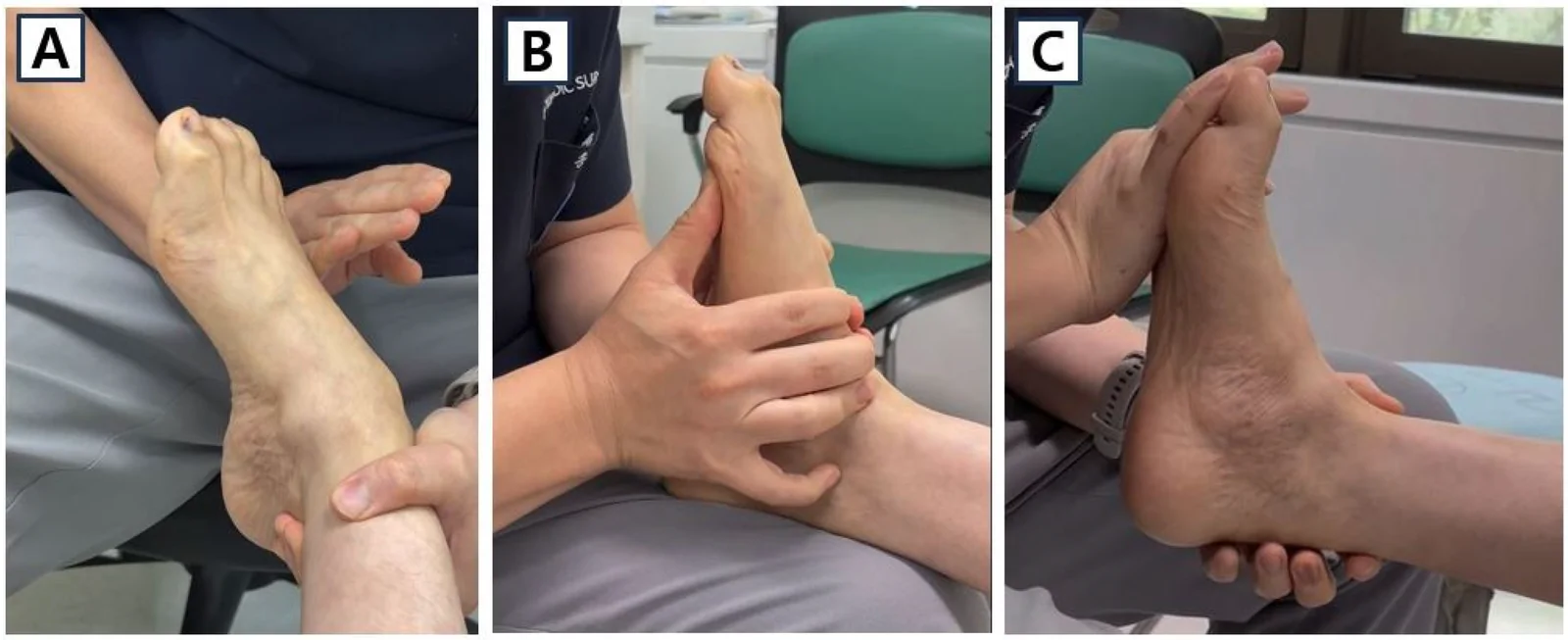
Physical examination of a patient with CMT-related foot deformity. Assessment of muscle strength of the (**A**) peroneus brevis and (**B**) peroneus longus. (**C**) Evaluation of ankle dorsiflexion and Achilles tendon tightness.

Among all aspects of the physical examination, the most critical step is an accurate assessment of the strength of each tendon controlling foot motion (Figure 2). The examiner should identify which tendons have been weakened by CMT and determine which functioning tendons are suitable candidates for tendon transfer to restore muscle balance. However, this evaluation is often challenging because patients with CMT frequently compensate for the weakness of an affected muscle by recruiting adjacent muscles, potentially masking the true extent of tendon dysfunction [1]. For example, when the tibialis anterior is weakened, preserved strength of the extensor hallucis longus and extensor digitorum longus may compensate for ankle dorsiflexion, creating the false impression that tibialis anterior function is intact. To minimize this compensatory effect, the metatarsophalangeal joints should be held in slight plantarflexion while the patient performs ankle dorsiflexion. This maneuver reduces recruitment of the toe extensors and allows a more accurate assessment of tibialis anterior strength [1]. Similarly, when the peroneal muscles are weakened, the extensor digitorum longus may generate a lateral dorsiflexion force during ankle dorsiflexion, creating the false impression that eversion strength is preserved. In this situation, eversion strength should be assessed with the ankle held in maximal inversion, which minimizes compensatory activation of the extensor digitorum longus and allows a more accurate evaluation of peroneal muscle function [3].

Assessment of peroneus longus strength is also essential for surgical planning, as it determines whether the tendon is suitable for transfer to the peroneus brevis [1]. However, accurate evaluation is often difficult because patients frequently compensate for first-ray plantarflexion by plantarflexing the ankle or hallux, or even dorsiflexing the hallux, rather than generating true peroneus longus contraction. To improve the accuracy of the examination, the examiner should place both thumbs beneath the first and fifth metatarsal heads and directly assess the plantarflexion force of the first metatarsal head, thereby minimizing compensatory movements and providing a more reliable assessment of peroneus longus function [3].

Although manual muscle testing remains practical and clinically important when evaluating potential donor tendons for transfer, it has inherent limitations. Quantitative measurements in patients with CMT have demonstrated considerable overlap between adjacent Medical Research Council muscle grades, indicating that ordinal manual grading may not fully reflect differences in actual muscle strength [15]. Hand-held dynamometry has also been used to objectively quantify ankle dorsiflexion and plantarflexion strength in patients with CMT and may provide complementary information when available [16]. Nevertheless, no single strength measurement should determine tendon-transfer selection, and muscle strength should be interpreted together with functional movement, gait pattern, deformity flexibility, and the availability of potential donor tendons [1,3].

Assessment of Achilles tendon contracture is also important, as equinus contracture is present in most patients with CMT-related foot deformity. The degree of contracture should be evaluated using the Silfverskiöld test and other clinical examinations. Because the Achilles tendon inserts slightly medial to the calcaneal axis, its contracture contributes not only to equinus but also to hindfoot varus, making Achilles tendon lengthening a common component of surgical correction [1,3].

### 4.3. Radiologic Study

**Figure 3:**
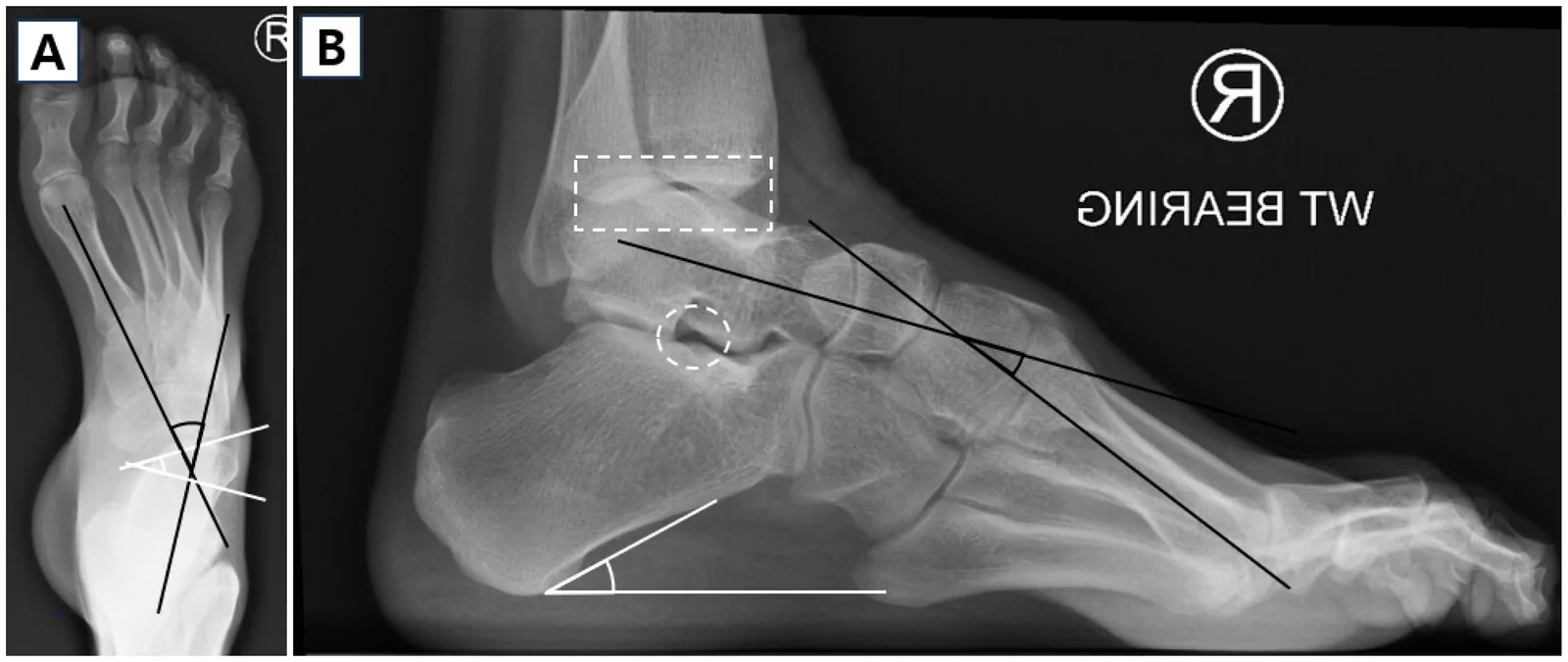
Radiographic measurements and characteristic signs used to evaluate CMT-related foot deformity. (**A**) Anteroposterior foot radiograph showing the axial Meary angle (black angle) and talonavicular coverage angle (white angle). (**B**) Lateral foot radiograph showing the sagittal Meary angle (black angle) and calcaneal pitch angle (white angle). The double talar dome sign (dashed rectangle) and sinus tarsi see-through sign (dashed circle) are also observed.

Weight-bearing radiographs remain the standard imaging modality for the evaluation of CMT cavovarus deformity (Figure 3). Recommended studies include weight-bearing anteroposterior (AP) and lateral foot radiographs, AP and lateral ankle radiographs, a hindfoot alignment view, and a full-length lower extremity scanogram [3,10]. On the AP foot radiograph, the axial Meary angle is formed by the longitudinal axes of the talus and first metatarsal and reflects forefoot and midfoot adduction. The talonavicular coverage angle is defined as the angle between a line connecting the medial and lateral margins of the articular surface of the talar head and a line connecting the medial and lateral margins of the proximal articular surface of the navicular [10]. On the hindfoot alignment view, hindfoot varus can be quantified using the hindfoot moment arm and hindfoot alignment angle. The hindfoot moment arm is measured as the shortest perpendicular distance between the longitudinal midtibial axis and the most inferior weight-bearing point of the calcaneus, whereas the hindfoot alignment angle is defined as the angle between the longitudinal axis of the tibial shaft and the longitudinal axis of the calcaneal tuberosity [9,10]. Interestingly, the calcaneal pitch may remain within the normal range in some patients with CMT despite a pronounced cavus foot. This paradoxical finding reflects the forefoot-driven nature of the deformity, in which marked midfoot supination compensates for calcaneal dorsiflexion, thereby masking an increased calcaneal pitch [17]. The hindfoot alignment view is used to evaluate hindfoot varus by measuring the hindfoot moment arm and hindfoot alignment angle [8,10]. Finally, AP ankle radiographs allow assessment of talar tilt and evaluation for degenerative changes in the ankle joint.

On the AP foot radiograph, the axial Meary angle is formed by the longitudinal axes of the talus and first metatarsal and reflects forefoot and midfoot adduction, whereas the talonavicular coverage angle reflects transverse-plane talonavicular incongruence. Hindfoot varus can be further quantified on the hindfoot alignment view using the hindfoot moment arm and hindfoot alignment angle.

Several additional radiographic findings are characteristic of CMT cavovarus deformity. Combined hindfoot varus, forefoot adduction, and the resulting external rotation of the hindfoot and ankle may produce the “see-through sign” of the sinus tarsi and the “double talar dome sign” on standard radiographs [10]. Metatarsal stacking is another characteristic finding, reflecting severe midfoot supination and inversion. Furthermore, repetitive lateral column overload caused by hindfoot varus and midfoot supination frequently increases stress across the fifth metatarsal, making stress fractures of the fifth metatarsal a relatively common radiographic finding in patients with CMT.

**Figure 4:**
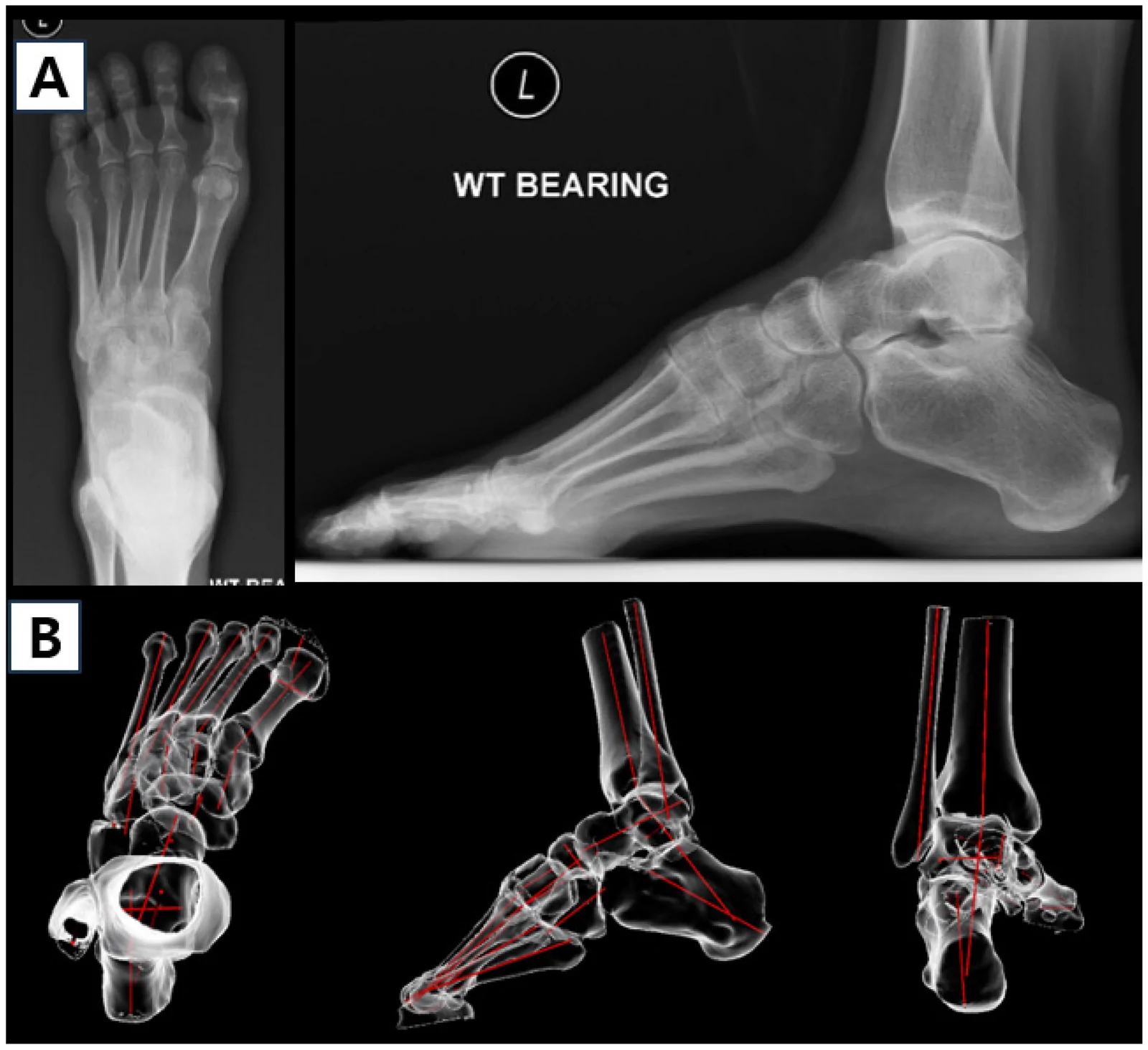
Images of CMT foot deformity. (**A**) Standing anteroposterior and lateral foot radiographs. (**B**) Segmental bone axes (red lines) identified using three-dimensional automated analysis software on weight-bearing CT images.

Computed tomography (CT) and magnetic resonance imaging (MRI) are selectively performed when additional anatomical information is required. Since CMT cavovarus deformity is a complex three-dimensional (3D) deformity, conventional weight-bearing radiographs and two-dimensional (2D) CT often fail to accurately characterize its morphology, resulting in limited interobserver reliability. More recently, weight-bearing CT (WBCT) combined with 3D automated analysis software (BoneLogic software 2.1) has enabled a more accurate and reproducible assessment of CMT foot deformities by quantitatively evaluating 3D alignment and rotational abnormalities (Figure 4) [11,18,19]. Song et al. [11] demonstrated the utility of WBCT combined with 3D automated analysis software for comprehensive evaluation of sagittal, axial, and coronal alignment in patients with CMT foot deformity before and after surgical reconstruction. These advanced imaging techniques provide valuable information for understanding the 3D nature of the deformity and assist in preoperative planning. However, they should be regarded as adjunctive tools rather than absolute guides for bony correction. The final decision regarding the type and extent of corrective osteotomy should be made only after all necessary soft-tissue releases have been completed, as the residual osseous deformity can be accurately assessed only after soft-tissue constraints have been eliminated [1].

Age may also influence the radiologic and clinical presentation of CMT foot deformity. A recent WBCT-based study comparing pediatric and adult patients with CMT found that adults demonstrated greater sagittal cavus and pain interference, whereas pediatric patients showed greater midfoot adduction and hindfoot varus [20]. These findings suggest that the clinical morphology observed in CMT may differ substantially according to age. However, because the study was cross-sectional and included unmatched symptomatic cohorts, the observed differences should not be interpreted as evidence of longitudinal progression from a pediatric to an adult deformity pattern. Longitudinal studies with serial imaging are required to clarify the natural evolution of CMT foot deformity.

Recent WBCT-based shape analyses have further demonstrated that the 3D morphology of CMT-related cavovarus deformity may differ according to disease subtype [21,22]. Requist et al. used a 14-bone statistical shape model and found that demyelinating CMT was associated with a more severe global cavovarus deformity and more pronounced hindfoot varus, whereas axonal CMT showed a relatively midfoot-centered pattern of deformity [21]. Peterson et al. similarly demonstrated intrinsic talar morphologic abnormalities in patients with both CMT1 and CMT2, including a flatter talar dome and a more medially oriented talar head and neck compared with controls [22]. These findings suggest that CMT-related cavovarus deformity reflects not only segmental malalignment but also intrinsic osseous morphologic changes and support the potential value of 3D imaging for subtype-specific structural characterization [21,22]. However, whether these morphologic differences should translate into different operative procedures or predict surgical outcomes remains unclear.

### 4.4. Gait and Biomechanical Assessment

Quantitative gait analysis can complement static clinical and radiographic assessment by objectively characterizing the functional consequences of CMT-related muscle weakness and foot deformity [23]. 3D gait studies have demonstrated several characteristic abnormalities, including foot drop during swing, impaired ankle plantarflexion, increased foot supination, and compensatory alterations at the knee and hip. The gait pattern may also vary according to the relative severity of dorsiflexor and plantar flexor weakness. Instrumented gait analysis may therefore help distinguish different functional phenotypes, quantify compensatory mechanisms, and provide objective outcome measures before and after treatment.

Beyond CMT-specific studies, quantitative biomechanical assessment has also demonstrated value in other congenital musculoskeletal disorders [24]. Glowinski et al. [24] used baropodometric gait analysis in a child with thrombocytopenia–absent radius syndrome and demonstrated that use of an upper-limb prosthesis altered plantar-pressure distribution, foot-ground contact time, and gait symmetry. Although these findings cannot be directly extrapolated to CMT, the study illustrates how instrumented gait and plantar-pressure analysis can identify compensatory loading patterns and treatment-related biomechanical changes that may not be apparent on static clinical or radiographic assessment. Similar quantitative approaches may provide complementary information in patients with CMT, particularly when evaluating the functional effects of orthotic or surgical interventions.

Nevertheless, availability of gait and biomechanical assessment remains limited, and should currently be regarded as an adjunct rather than a mandatory component of routine surgical decision-making.

## 5. Treatment

### 5.1. Conservative Treatment

In the early stage of CMT, when the deformity is mild and the joints remain flexible, custom shoe orthoses and bracing are the preferred initial treatment options. Orthoses incorporating a lateral wedge and a recessed area beneath the first metatarsal head can accommodate first-ray plantarflexion and hindfoot varus, helping to achieve a plantigrade foot. As the deformity progresses, an ankle–foot orthosis (AFO) can provide improved control of hindfoot alignment. Approximately 20% of patients with CMT present with profound muscle weakness below the knee but without severe structural foot deformity. In these patients, a ground-reaction ankle–foot orthosis (GRAFO) is recommended to improve gait by compensating for the loss of distal muscle function [3]. Recent advances in orthotic design and manufacturing have further enhanced functional ambulation even in patients with advanced CMT deformity. Accordingly, ankle arthrodesis should be avoided whenever possible in the absence of severe deformity, as preserving ankle motion allows patients to derive greater benefit from current orthotic technologies [3].

Rehabilitation represents another important component of nonoperative management. A systematic review of exercise interventions in CMT reported potential improvements in strength, functional activity, and physiological measures, although the number and methodological quality of available studies were limited and the optimal exercise modality and intensity remain uncertain [25]. Therefore, rehabilitation programs should be individualized and may incorporate strengthening, balance and proprioceptive training, stretching, and aerobic conditioning according to the patient’s weakness, fatigue, joint mobility, and functional requirements [25].

### 5.2. Operative Treatment

#### 5.2.1. Timing of Surgery

The optimal timing for surgical intervention in patients with CMT disease remains controversial. Many patients experience progressive foot deformity and muscle weakness during adolescence, leading to increasing difficulty with daily activities and ambulation, eventually requiring orthotic support and consideration of surgery. Conservative management with braces should remain the first-line treatment as long as a plantigrade foot can be maintained while wearing the brace. However, when orthotic management no longer provides adequate functional improvement or patients continue to experience difficulty with ambulation and daily activities, surgical intervention should be considered. Although some patients may still require brace use after surgery, correction of the deformity often improves brace fitting and functional ambulation. Therefore, patients with progressive cavovarus deformity or foot drop should be referred to a foot and ankle specialist for timely surgical evaluation [3].

Although the ideal timing of surgery has not been clearly established, prolonged untreated deformity tends to progress, resulting in increasing soft-tissue contracture, skin ulceration, joint instability, and degenerative arthritis, all of which make reconstruction more complex. Based primarily on expert consensus rather than comparative clinical trials, earlier surgical treatment before the deformity becomes rigid is generally advocated when progressive symptoms cannot be adequately controlled with orthotic treatment [1,3]. However, whether earlier joint-sparing reconstruction alters the natural history of CMT deformity or reduces the subsequent need for arthrodesis has not been established in controlled longitudinal studies [3].

#### 5.2.2. Joint-Sparing Surgery

When conservative treatment fails to provide satisfactory symptom relief or functional improvement, surgical intervention should be considered. The primary goal of surgical treatment is to restore and maintain a balanced plantigrade foot, thereby improving gait stability and walking efficiency. Recently, the AOFAS recommended that joint-sparing reconstruction should be considered whenever feasible in patients with CMT-related cavovarus deformity. In contrast, joint fusion procedures, such as subtalar arthrodesis or ankle arthrodesis, should be reserved for patients with painful arthritic joints or rigid deformities that cannot be adequately corrected with joint-preserving procedures [3].

Nevertheless, the available evidence does not support a single standardized surgical strategy for all patients with CMT-related cavovarus deformity. Most published surgical studies include relatively small and heterogeneous patient populations, with considerable variation in age, disease severity, deformity pattern, muscle imbalance, and combinations of operative procedures [16,26,27]. Recent 3D morphologic studies have also suggested differences between demyelinating and axonal CMT, including more pronounced hindfoot varus in demyelinating disease and a relatively midfoot-centered deformity in axonal disease [21,22]. Although these findings may eventually contribute to more individualized treatment planning, current evidence is insufficient to establish genotype- or subtype-specific surgical algorithms. Therefore, surgical decision-making remains primarily phenotype-driven and should be individualized according to deformity flexibility, the location and severity of osseous deformity, individual muscle-tendon function, joint degeneration, patient age, and functional requirements [1,3]. The stepwise reconstruction described below represents one practical joint-sparing strategy rather than a universally established operative protocol.

Joint-sparing procedures generally consist of soft-tissue release, corrective osteotomy, and tendon transfer. The sequence of reconstruction is critical: release of all contracted or deforming soft tissues should be performed before corrective osteotomy [1,3]. Because motion across the hindfoot joints is interdependent, all soft-tissue contractures must be adequately addressed before bony correction is attempted. For example, release of a contracted tibialis posterior tendon can reduce foot supination, hindfoot varus, and midfoot adduction. Only after the foot position has been optimized through soft-tissue release can osteotomy and bony realignment be performed accurately and effectively. Incomplete soft-tissue release before corrective osteotomy may result in undercorrection or recurrent deformity. Conversely, performing corrective osteotomy before adequate soft-tissue release may increase the risk of overcorrection [1].

Song et al. [11] evaluated the effectiveness of joint-sparing surgery in 29 patients with CMT using automated analysis of preoperative and postoperative WBCT images. The authors demonstrated that joint-sparing surgery effectively corrected deformities in the sagittal, axial, and coronal planes of the CMT foot. They further suggested that restoration of talonavicular alignment through comprehensive soft-tissue release around the talonavicular joint may represent an important component of successful deformity correction. In this series, patients underwent a mean of 7.8 surgical procedures (range, 5–10), with the specific combination of procedures individualized according to the pattern of deformity and the degree of muscle imbalance. The most frequently performed procedures, in sequential order, included percutaneous Achilles tendon triple hemisection (79.3%), posterior tibialis release (82.8%), talonavicular joint and spring ligament release (89.7%), percutaneous flexor digitorum longus tenotomy (62.1%), midfoot plantar fascia release (93.1%), peroneus longus tendon release (93.1%), calcaneal osteotomy (82.8%), transfer of the peroneus longus to the peroneus brevis (93.1%), first metatarsal dorsiflexion closing wedge osteotomy (86.2%), and posterior tibialis tendon transfer (82.8%) [11].

① Soft tissue release

**Figure 5:**
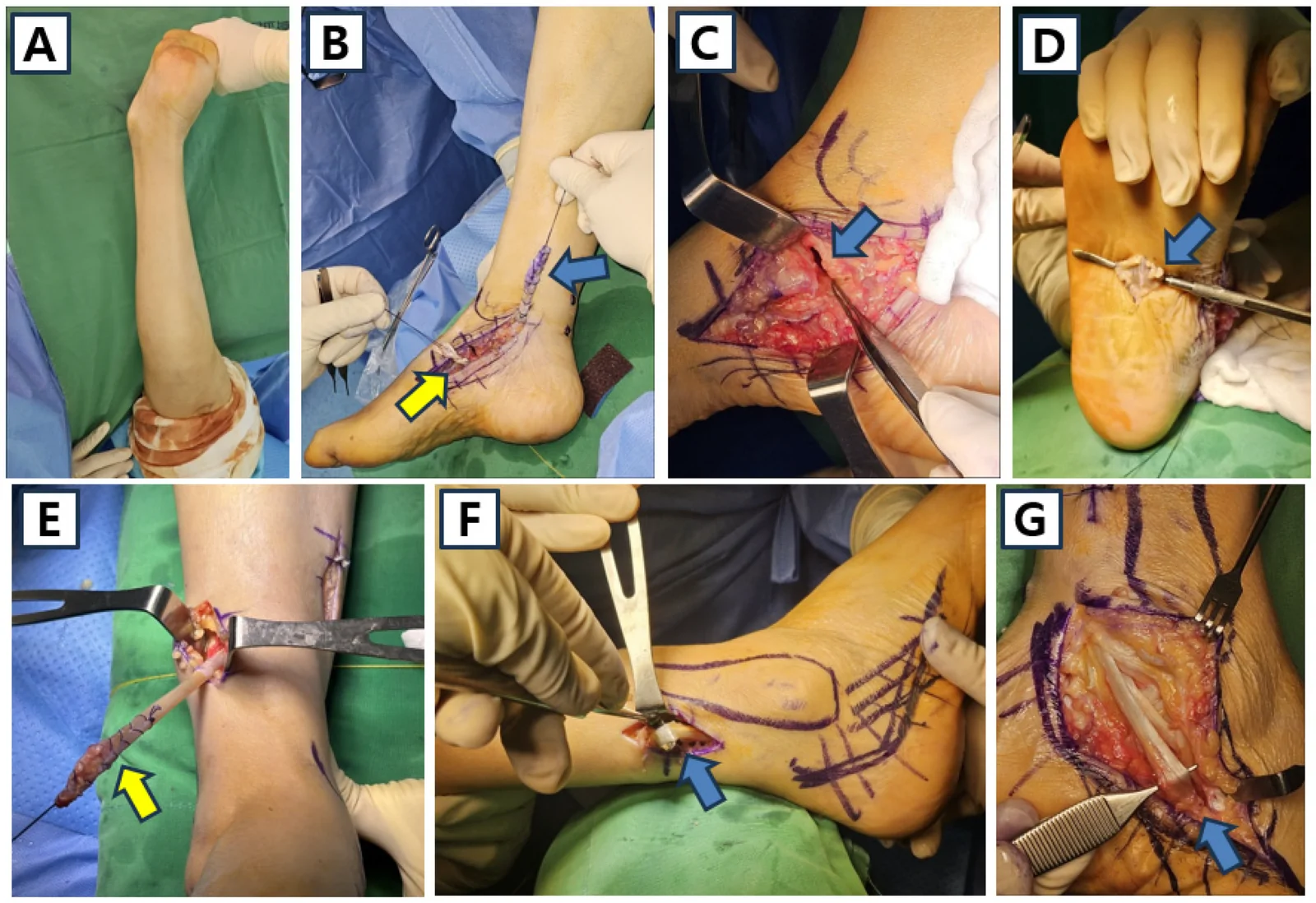
Photographs showing the operative steps of soft tissue release during CMT foot reconstruction. Soft tissue release procedures were performed before corrective osteotomies and tendon transfers. (**A**) Preoperative hindfoot varus deformity. (**B**) Release of the posterior tibial tendon (blue arrow) and flexor hallucis longus tendon (yellow arrow). The flexor hallucis longus tendon was released for subsequent transfer to the peroneus longus tendon. (**C**) Release of the talonavicular joint capsule (blue arrow). (**D**) Plantar fascia release (blue arrow) at the midfoot. (**E**) Passage of the posterior tibial tendon (yellow arrow) from the posteromedial to the anterolateral aspect of the lower leg through the interosseous membrane. (**F**) Passage of the flexor hallucis longus tendon (blue arrow) from the medial to the posterolateral aspect of the ankle. (**G**) Release of the peroneus longus tendon (blue arrow).

Joint-sparing reconstruction should be performed in a stepwise manner, beginning with comprehensive soft tissue release followed by corrective osteotomy (Figure 5).

Correction of the equinus deformity begins with a percutaneous Achilles tendon lengthening using the triple hemisection technique (Hoke procedure), with the proximal and distal incisions made medially, when Achilles tendon contracture is present. In addition to correcting equinus, Achilles tendon lengthening facilitates subsequent correction of hindfoot varus. In the CMT foot, release of the tibialis posterior tendon is a critical step in correcting varus deformity because the tendon remains relatively overpowered compared with the weakened peroneus brevis. Through a longitudinal medial incision, the tibialis posterior tendon is released from its insertions on the navicular and medial cuneiform. During this step, it is essential to preserve the distal tendon stump attached to the medial cuneiform as long as possible to facilitate subsequent tendon transfer. In younger pediatric patients, tibialis posterior tendon lengthening may be performed instead of complete release in selected cases [1]. The next step is release of the contracted talonavicular joint, which is considered the most critical component of cavovarus deformity in the CMT foot [11,28]. Additional release of the contracted spring ligament is performed as needed. In contrast, release of the subtalar joint should be avoided because it may result in overcorrection of the hindfoot varus deformity [1].

A longitudinal incision is then made over the distal one-third of the medial leg. After incising the deep posterior compartment fascia and retracting the flexor digitorum longus tendon, the previously released tibialis posterior tendon is delivered through the medial incision. A second longitudinal incision is created over the anterolateral aspect of the distal leg, slightly distal to the medial incision. Following incision of the anterior compartment fascia and retraction of the tibialis anterior tendon, the tibialis posterior tendon is transferred from the medial side to the anterior compartment through the interosseous membrane and retrieved through the anterolateral incision.

In patients with claw toe deformities secondary to intrinsic muscle imbalance, percutaneous flexor digitorum longus tenotomy of the second through fifth toes is performed. A transverse incision is then made over the medial plantar aspect of the midfoot to release the plantar fascia. Next, a curvilinear lateral incision extending from the posterior aspect of the calcaneus toward the base of the fifth metatarsal is made to expose the peroneus longus and peroneus brevis tendons. The peroneus longus tendon is transected just proximal to the os peroneum or at the level of the cuboid tunnel to correct the plantarflexed first metatarsal and is subsequently used for transfer to the peroneus brevis to augment eversion strength. Adequate release of all contracted soft tissues is essential to optimize foot alignment before corrective osteotomy, thereby allowing accurate and effective osseous correction.

② Corrective osteotomy and tendon transfer

**Figure 6:**
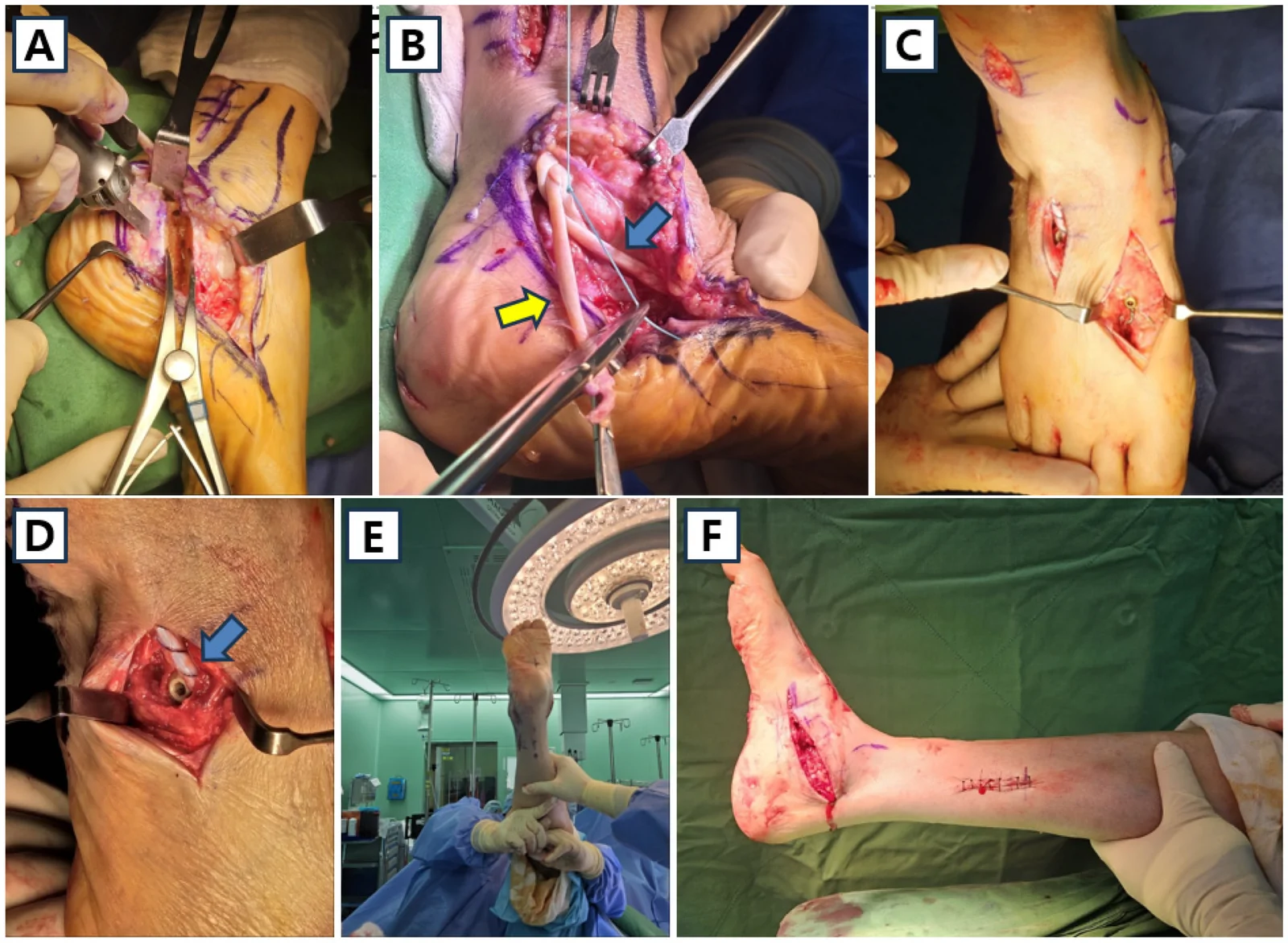
Photographs showing the operative steps of osteotomy and tendon transfer during CMT foot reconstruction. (**A**) Lateral closing-wedge osteotomy of the calcaneus. (**B**) Transfer of the flexor hallucis longus tendon (yellow arrow) to the peroneus brevis tendon (blue arrow). (**C**) Basilar dorsal closing-wedge osteotomy of the first metatarsal. (**D**) Transfer of the posterior tibial tendon (blue arrow) to the lateral cuneiform. (**E**,**F**) Neutral alignment of the foot and ankle after reconstruction.

After transection of the peroneus longus tendon, the sural nerve is carefully retracted, and a calcaneal osteotomy is performed through the posterior calcaneal tuberosity. The osteotomy is created at approximately 45° within 1 cm anterior to the insertions of both the Achilles tendon and the plantar fascia. The better hindfoot correction is achieved with a lateral closing wedge osteotomy, lateral translation, and valgus rotation of the calcaneal tuberosity (Figure 6) [11,29,30]. This 3D calcaneal osteotomy is performed not only to correct hindfoot varus but also to restore the intrinsic deformity of the calcaneus, which characteristically assumes a C-shaped morphology in patients with CMT [29]. An et al. [30] compared the outcomes of various calcaneal osteotomy techniques for the correction of cavovarus deformity using 3D-printed calcaneal models and demonstrated that a combined osteotomy consisting of a lateral closing wedge, lateral translation, and valgus rotation provided the most effective correction of the deformity. Release of the plantar fascia attached to the plantar aspect of the posterior calcaneal tuberosity facilitates mobilization and correction of the calcaneal fragment during osteotomy. After osteotomy and reduction, the calcaneus is temporarily stabilized with two Kirschner wires, and correction of the hindfoot varus is confirmed visually. Definitive fixation is then achieved using two cannulated screws inserted from posterior to anterior.

Following calcaneal correction, tendon transfer is performed to augment hindfoot eversion. With the subtalar joint held in eversion, the previously harvested peroneus longus tendon is transferred to the peroneus brevis using a Pulvertaft weave at the distal fibula. If the peroneus longus is substantially weakened (Medical Research Council muscle strength grade < 3) and unsuitable for transfer, the flexor hallucis longus or flexor digitorum longus tendon may be used as an alternative donor tendon for transfer to the peroneus brevis [11].

To correct plantarflexion of the first metatarsal, a proximal first metatarsal dorsal closing wedge osteotomy is performed, followed by fixation using a tension band wiring. Depending on the degree of correction required, fixation may alternatively be achieved with a plate or staple. The osteotomy should be performed as proximally as possible in the first metatarsal while preserving the plantar cortex as a hinge, and a 5–7 mm dorsal wedge of bone is typically resected. In pediatric patients with an open first metatarsal physis, a plantar opening wedge osteotomy of the medial cuneiform using an allograft may be performed as an alternative to first metatarsal osteotomy [1].

Finally, the lateral cuneiform is identified under C-arm fluoroscopic guidance, and a longitudinal dorsal incision is made over the bone. The transferred tibialis posterior tendon is retrieved from the anterolateral leg incision and passed beneath the extensor retinaculum through the anterior compartment using a tendon passer before being delivered through the dorsal incision over the lateral cuneiform. With the ankle held in a neutral position, the tendon is secured to the lateral cuneiform using a tenodesis interference screw. Care should be taken to avoid overtensioning the transferred tendon, which may result in excessive ankle dorsiflexion. At the completion of the procedure, approximately 10° of ankle plantarflexion should remain available [1]. If the tibialis posterior tendon is too weak to serve as a donor tendon, alternative tendon transfers using the extensor tendons (extensor hallucis longus and extensor digitorum longus) or flexor tendons (flexor hallucis longus and flexor digitorum longus) to the midfoot may be considered [1,31].

#### 5.2.3. Joint Fusion Surgery

**Figure 7:**
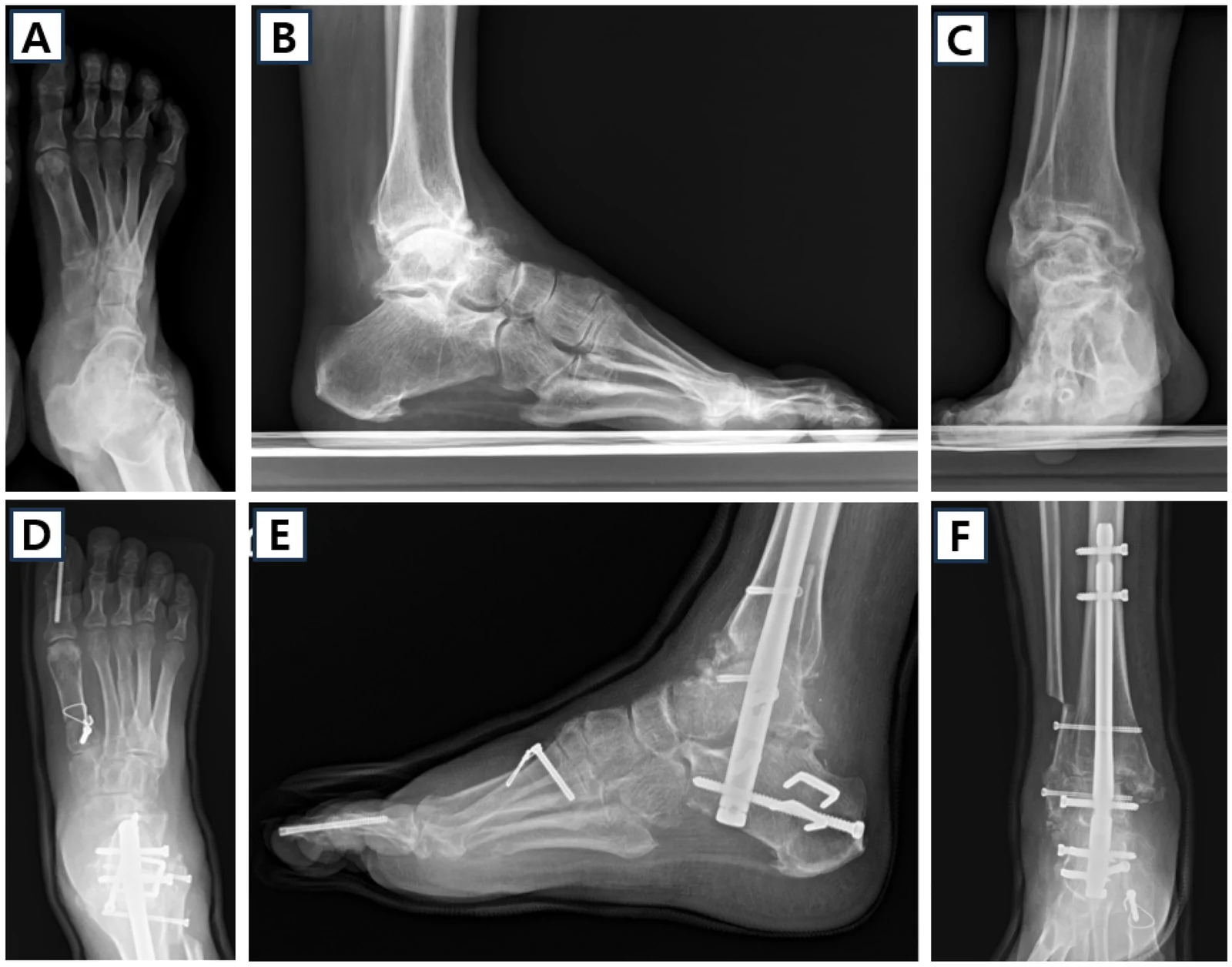
Preoperative and postoperative radiographs of the right foot and ankle in a 40-year-old woman with CMT who underwent joint fusion surgery. Compared with (**A**–**C**) the preoperative radiographs, (**D**–**F**) the postoperative radiographs demonstrate restoration of neutral foot and ankle alignment following arthrodesis.

When no suitable tendon is available for transfer to augment eversion, or when severe deformity cannot be adequately corrected with osteotomy and tendon transfer alone, or in the presence of painful arthritic joints, subtalar, triple, tibiotalar, or tibiotalocalcaneal fusion is recommended (Figure 7) [1]. Current consensus and expert recommendations generally favor joint preservation when feasible, particularly in younger patients, whereas arthrodesis is commonly considered for rigid, noncorrectable deformity or painful degenerative joints [1,3]. However, comparative evidence demonstrating the long-term superiority of one strategy over another remains limited. Arthrodesis is preferably combined with soft tissue release, corrective osteotomy, and tendon transfer to optimize deformity correction and restore muscle balance [3].

If the tibialis posterior tendon has sufficient strength for transfer to the dorsum of the foot but no suitable tendon is available to augment eversion because of weakness of the peroneal tendons or flexor hallucis longus, subtalar arthrodesis is a useful option for enhancing hindfoot stability [1]. In patients with severe midfoot deformity associated with talonavicular joint dislocation or subluxation, talonavicular and calcaneocuboid arthrodesis may be required. When triple arthrodesis is performed, concomitant calcaneal osteotomy is often necessary, and two 6.5-mm cannulated screws can be used to achieve fixation of both the corrective calcaneal osteotomy and the subtalar arthrodesis. In patients with varus ankle deformity, lateral ligament repair should be considered as the initial surgical option, whereas tibiotalar or tibiotalocalcaneal fusion should be reserved for cases in which satisfactory correction cannot be achieved.

A summary of treatment strategies for CMT foot deformity is provided in Table 1.

#### 5.2.4. Postoperative Care

Immediately after surgery, the surgical wound is dressed, and a short leg cast is applied whenever possible before the patient leaves the operating room. Sutures are typically removed 2 weeks postoperatively. Following joint-sparing reconstruction, patients are maintained in a short leg cast with strict non-weight-bearing for 6 weeks, followed by weight-bearing as tolerated in a controlled ankle motion (CAM) boot for an additional 2 weeks. Regular shoe wear is generally permitted after 8 weeks, at which point physical therapy and rehabilitation are initiated. Because CMT foot deformity is frequently bilateral, surgery on the contralateral foot can be scheduled at least 8 weeks after the initial procedure.

Following arthrodesis, patients remain non-weight-bearing in a short leg cast for 6 weeks, followed by protected weight-bearing in the cast for an additional 4 weeks. A CAM boot is introduced after 10 weeks, followed by physical therapy and rehabilitation.

After adequate soft-tissue and osseous healing has been achieved, postoperative rehabilitation should be individualized according to the procedures performed and the patient’s residual neuromuscular function. Rehabilitation may include progressive range-of-motion exercises, strengthening of preserved and transferred muscle-tendon units, balance and proprioceptive training, gait retraining, and reassessment of orthotic requirements [25]. However, evidence specifically comparing postoperative rehabilitation protocols after CMT foot reconstruction is lacking, and the optimal intensity and progression of rehabilitation remain to be established.

Patients should be followed for at least 1 year after reconstructive surgery with serial clinical and radiographic evaluations to monitor healing, alignment, and functional outcomes.

## 6. Prognosis

**Figure 8:**
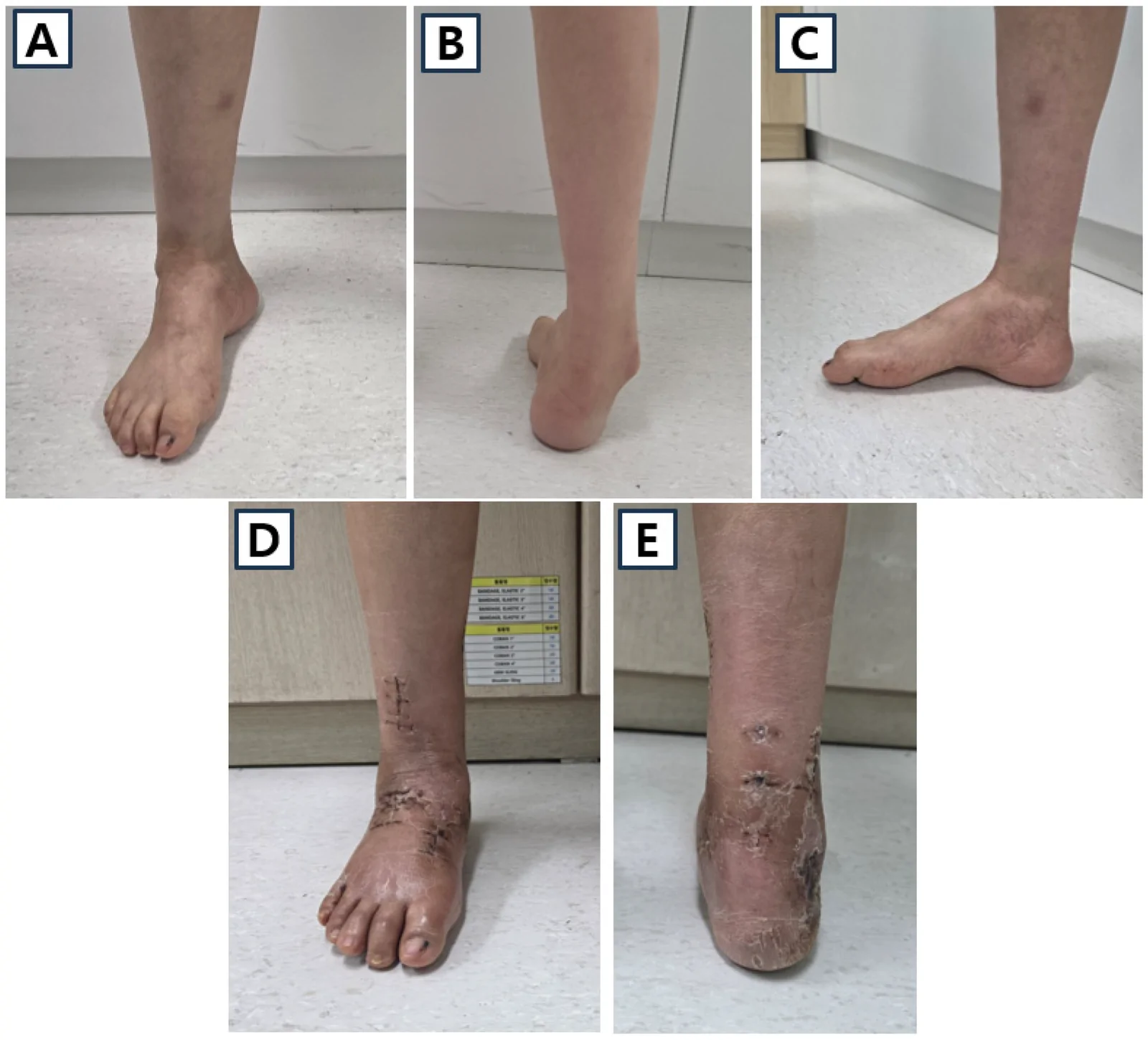
Standing photographs showing the right foot before (**A**–**C**) and after (**D**,**E**) joint-sparing surgery in CMT foot deformity. The 6-week postoperative images demonstrate satisfactory correction of the cavovarus deformity and restoration of neutral foot alignment.

Haupt et al. [12] conducted a retrospective study of 95 feet in 64 patients who underwent surgical treatment for CMT-related foot deformity and evaluated changes in the Patient-Reported Outcome Measurement Information System (PROMIS) scores over a mean follow-up period of 21 months. The study demonstrated significant postoperative improvements in all three PROMIS domains, including physical function, pain interference, and mental health/depression, compared with preoperative scores (Figure 8). The authors also compared clinical outcomes between patients who underwent joint-sparing reconstruction (37 patients) and those treated with arthrodesis (27 patients). Although both groups experienced postoperative clinical improvement, the arthrodesis group demonstrated significantly lower physical function than the joint-sparing group both before and after surgery [12].

Prospective evidence also supports meaningful functional benefits after surgical correction. Ramdharry et al. prospectively evaluated 25 adults with CMT undergoing foot surgery and found significant improvements in foot alignment, pain, callosities, and quality of life at 1 year, with these benefits maintained for up to 4 years [27]. However, surgery did not significantly improve fatigue, balance, or overall CMT examination scores, emphasizing that reconstructive surgery addresses the mechanical consequences of the disease rather than the underlying progressive neuropathy.

Long-term follow-up data are available from Ward et al., who evaluated 41 feet after joint-preserving reconstruction at a mean follow-up of 26.1 years [26]. Correction of the cavus deformity was generally maintained, although recurrent hindfoot varus was common [26]. Seven patients involving eight feet underwent additional foot or ankle operations, and moderate-to-severe osteoarthritis was observed in 11 feet; nevertheless, none of the patients subsequently required triple arthrodesis. These findings suggest that joint-sparing reconstruction may provide durable correction in appropriately selected patients while also emphasizing the possibility of recurrent deformity, degenerative changes, and additional surgery during long-term follow-up.

Taken together, these studies consistently suggest symptomatic and functional benefit after appropriately selected reconstruction, but their observational designs, limited sample sizes, heterogeneous procedures, and absence of randomized or well-matched comparative cohorts preclude definitive conclusions regarding the superiority of any specific surgical strategy.

## 7. Limitations of Current Evidence and Future Directions

The current evidence regarding orthopedic management of CMT-related foot deformity has several important limitations. Most surgical studies consist of retrospective case series or relatively small prospective cohorts, and substantial heterogeneity exists in patient age, disease severity, genotype, surgical technique, rehabilitation protocols, duration of follow-up, and outcome measures [16,26,27]. Consequently, direct comparison between operative strategies remains difficult, and high-quality evidence defining the optimal timing of surgery or superiority of one reconstructive strategy over another is lacking.

Interpretation of the surgical literature is further complicated by important differences in study design and treatment indication [11,12,16,26,27]. Most available studies are uncontrolled retrospective series, while only a small number of prospective observational studies have evaluated postoperative outcomes [12,26,27]. In addition, surgical procedures are rarely performed in isolation; patients frequently undergo individualized combinations of soft-tissue release, tendon transfer, and multiple osteotomies, making it difficult to attribute improvement to any single procedure [11,16,26]. Comparisons between joint-sparing reconstruction and arthrodesis are also vulnerable to confounding by indication, because arthrodesis is generally selected for patients with rigid deformity or painful degenerative joints, whereas joint-sparing reconstruction is considered when adequate correction can still be achieved [3,12]. Accordingly, differences in postoperative outcomes between surgical groups should not be interpreted as evidence of superiority of one strategy without appropriately controlled comparative studies.

Another important unresolved issue is the potential influence of CMT subtype on orthopaedic treatment. Recent 3D imaging studies demonstrate differences in global foot morphology between demyelinating and axonal CMT and intrinsic talar abnormalities in both CMT1 and CMT2 [21,22]. However, these studies primarily characterize morphology rather than clinical outcomes, and there is currently insufficient evidence to determine whether subtype-specific morphologic patterns should lead to different osteotomies, tendon transfers, or arthrodesis strategies.

Future multicenter prospective studies should incorporate detailed genetic characterization, standardized 3D imaging, quantitative gait assessment, objective muscle-strength evaluation, patient-reported outcomes, and long-term follow-up. Such studies may help determine whether genotype or subtype independently influences deformity progression, optimal timing of intervention, recurrence, and long-term surgical outcomes.

## 8. Conclusions

A substantial proportion of patients with CMT develop cavovarus foot deformity that can substantially impair gait and function. Conservative treatment remains the initial approach for flexible deformities that can be adequately accommodated with orthotic management. When symptoms and functional limitations persist despite nonoperative treatment, surgical reconstruction may be considered with the goal of restoring a stable, plantigrade foot and improving mechanical function. Current consensus favors joint-sparing surgery using individualized combinations of soft-tissue release, osteotomy, and tendon transfer when adequate correction is feasible, whereas arthrodesis is generally considered for rigid, irreversible deformity or painful degenerative joints.

However, these recommendations are supported predominantly by expert consensus, retrospective case series, and a limited number of prospective observational studies. Substantial heterogeneity in patient characteristics, operative techniques, follow-up duration, and outcome measures precludes definitive conclusions regarding the optimal timing of surgery or superiority of a specific reconstructive strategy. Future prospective multicenter studies integrating genetic characterization, standardized 3D imaging, quantitative biomechanical assessment, and patient-reported outcomes are needed to establish more individualized and evidence-based treatment strategies.

## Figures and Tables

**Table 1:** A summary of treatment strategies for CMT foot deformity.

Treatment Strategy	Indications	Advantages	Limitations	Expected Outcomes
Conservative treatment	Mild or flexible deformity; acceptable function with orthosis	Preserves joint motion; nonoperative; adaptable to disease progression	Does not correct fixed deformity; variable response depending on weakness and brace tolerance	Improved stability and gait; maintenance of function
Joint-sparing reconstruction	Progressive symptomatic but surgically correctable deformity	Preserves joint motion; corrects structural deformity and muscle imbalance	Technically complex; often requires multiple procedures; risk of recurrence	Improved alignment, pain relief, and function; durable correction in selected patients
Arthrodesis	Rigid deformity, painful arthritis, or failed reconstruction	Strong correction and high mechanical stability	Loss of joint motion; risk of nonunion and hardware complications;	Reliable pain relief and plantigrade foot alignment in severe cases

## Data Availability

The data presented in this study are available in the article.
